# The pharmacokinetic challenge of voriconazole therapy for cerebral aspergillosis in patients treated with ibrutinib

**DOI:** 10.1186/s13054-019-2385-x

**Published:** 2019-03-12

**Authors:** Rémy Nyga, Laura Simon, Taieb Chouaki, Caroline Delette, Youssef Bennis, Cedric Joseph, Jean-Pierre Marolleau, Michel Slama, Elie Zogheib, Julien Maizel

**Affiliations:** 1 Service de Médecine Intensive Réanimation, Rond point du Pr Cabrol, 80054 Amiens CEDEX 1, France; 2 Department of Clinical Hematology and Cellular Therapy, Amiens University Hospital, Amiens, France; 3 Medical Parasitology and Mycology Department, Amiens University Hospital, Amiens, France; 4 Laboratory of Pharmacology and Toxicology, Department of Clinical Pharmacology, Amiens University Hospital, Amiens, France; 5 MP3CV-EA 7517, Picardy Jules Verne University, Amiens, France; 6 Department of Infectious Diseases, Amiens University Hospital, Amiens, France; 7 AGIR: Microbiology Research Unit, EA4294, Equipe AGIR, Université de Picardie Jules Verne, Amiens, France; 8 EA 4666, Picardy Jules Verne University, Amiens, France

Ibrutinib is a new Bruton’s tyrosine kinase inhibitor approved for the management of chronic lymphocytic leukemia (CLL) that has recently been associated with an increasing number of cases of invasive aspergillosis (IA). Ghez et al. reported 33 patients with invasive fungal infections, corresponding to IA in 27/33 with cerebral aspergillosis in 40% of these cases [1]. Voriconazole (VRCZ) is the first-line treatment for IA including central nervous system (CNS) infection due to its good penetration across the blood-brain barrier. However, VRCZ requires therapeutic drug monitoring to ensure effective therapy. In the case reported here, CNS aspergillosis was responsible for brain edema requiring corticosteroids. However, corticosteroids have been very recently reported to be a new cause of rapid VRCZ metabolism, inducing low plasma VRCZ concentrations and therefore limited efficacy [2].

A 69-year-old man with a history of refractory CLL treated with ibrutinib was admitted to the ICU with ARDS (acute respiratory distress syndrome) secondary to invasive pulmonary aspergillosis (Fig. 1a–c). Therefore, intravenous VRCZ was initiated and ibrutinib was stopped. Three weeks later, brain MRI was performed following the onset of neurological signs and revealed bilateral nodular lesions consistent with cerebral IA associated with brain edema requiring corticosteroids (methylprednisolone) (Fig. 1d). Corticosteroid therapy significantly reduced brain edema and improved clinical symptoms. However, several days after, a new elevation of galactomannan (GM) antigen was observed in serum and BAL fluid despite VRCZ therapy. Elevated galactomannan was associated with a marked decrease of plasma VRCZ concentrations, requiring an increase of the VRCZ dosage (Fig. 2). Corticosteroids were stopped 2 weeks later, followed by a marked increase of plasma VRCZ concentrations and negative GM antigen (Fig. 2).

**Fig. 1:**
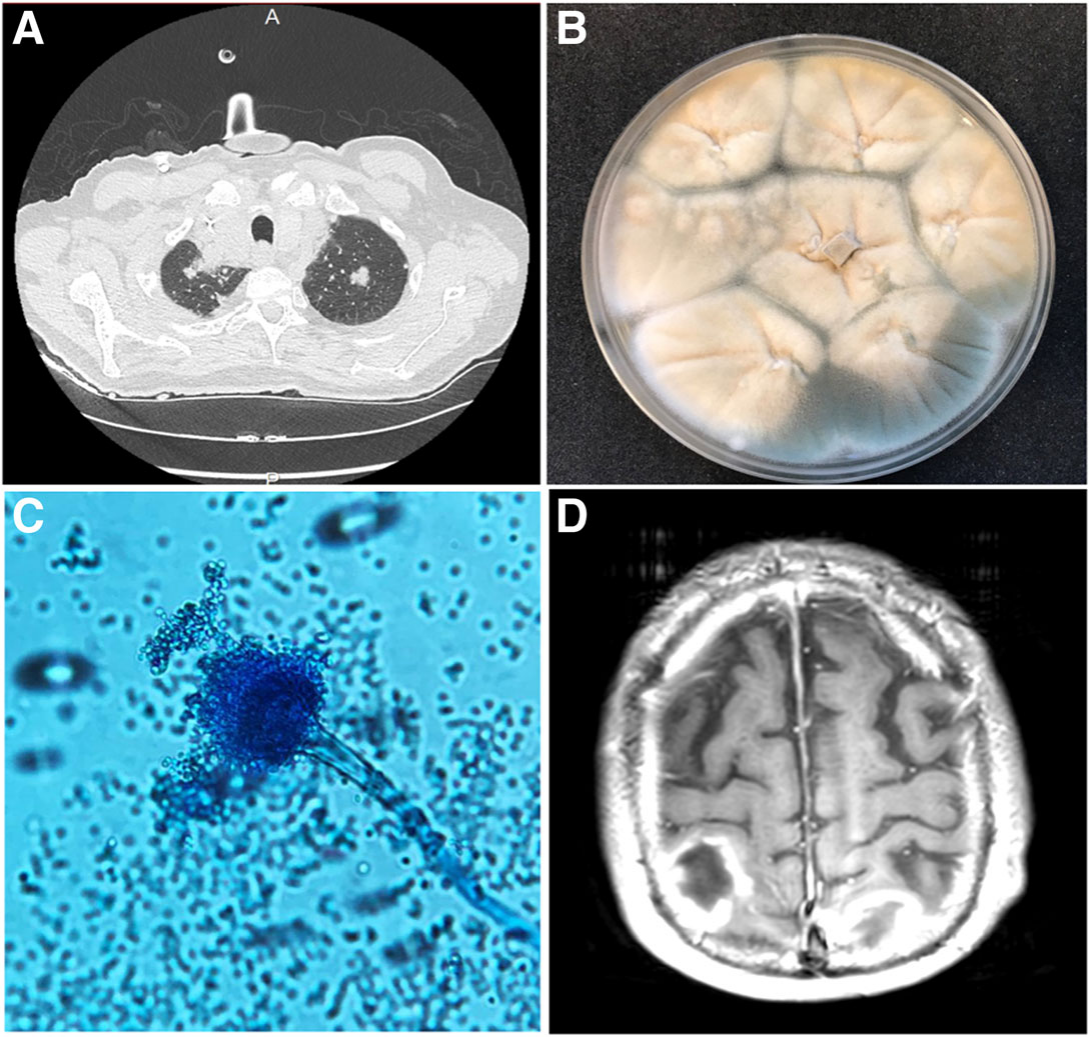
Diagnosis of IPA. **a** Axial chest CT scan showing bilateral upper lobe lung nodules; **b** BAL culture on the 8th day showing whitish and greenish powdery, granular growth of *Aspergillus*; **c** Microscopic examination with Lactophenol Cotton Blue staining showing *Aspergillus fumigatus* conidiophores and free conidia (× 400); **d** Brain MRI revealing multiple bilateral nodular lesions, gadolinium-enhanced T1-weighted transverse brain MRI

**Fig. 2:**
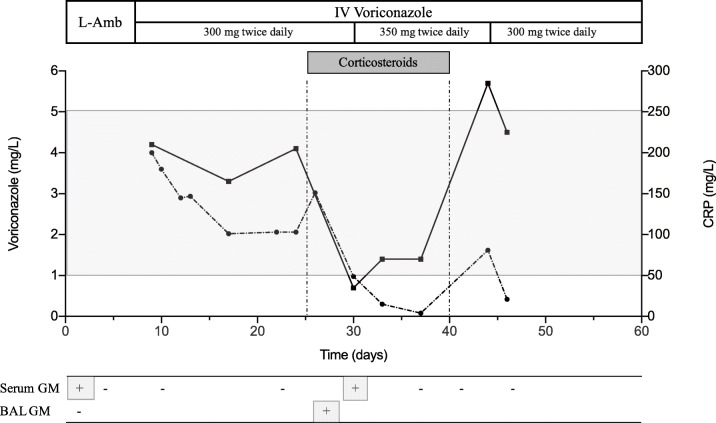
Time-course of voriconazole and C-reactive protein (CRP) concentrations in the presence and absence of corticosteroids. The *X*-axis corresponds to time (days). The left *Y*-axis corresponds to plasma voriconazole concentration (mg/L) and the right *Y*-axis corresponds to serum CRP levels (mg/L). Plasma voriconazole and CRP concentrations are represented by a black line and a dotted line, respectively. The *gray shaded area* indicates the therapeutic range of voriconazole (between 1 and 5 mg/L). The lower part of the figure corresponds to the course of serum galactomannan antigen (*serum GM*) and bronchoalveolar lavage galactomannan antigen (*BAL GM*). NB: L-Amb: liposomal amphotericin-B

The reduction of plasma VRCZ levels is a poorly known effect by physicians associated with concomitant corticosteroid therapy, as corticosteroids are potent inducers of CYP2C19 and CYP3A in humans, both of which are implicated in VRCZ metabolism [3]. Also inflammation, as reflected by the C-reactive protein (CRP) concentration, also increases plasma VRCZ concentrations as a result of decreased metabolism [2]. Corticosteroid therapy can therefore lead to a rapid decrease of plasma VRCZ concentrations.

This situation could become increasingly frequent in view of the growing number of cases of CNS aspergillosis observed in patients treated with ibrutinib. Physicians must therefore be aware of the drug-drug interaction between VRCZ and corticosteroids for cytochrome P450, which can lead to decreased plasma VRCZ concentrations and therefore limited efficacity against the *Aspergillus*.

## Abbreviations

ARDS: Acute respiratory distress syndrome
BAL: Bronchoalveolar lavage
CLL: Chronic lymphocytic leukemia
CNS: Central nervous system
CS: Corticosteroids
CYP: Cytochrome P450
CRP: C-reactive protein
IA: Invasive aspergillosis
ICU: Intensive care unit
MRI: Magnetic resonance imaging
VRCZ: Voriconazole
